# Prevention of Parent to Child Transmission (PPTCT) Program Data in India: An Emerging Data Set for Appraising the HIV Epidemic

**DOI:** 10.1371/journal.pone.0048827

**Published:** 2012-11-15

**Authors:** Sema K. Sgaier, Radhay S. Gupta, Raghuram Rao, Ajay Gaikwad, Sonali Harangule, Suvidha Dhamne, Sateesh Gowda, Sylvia Jayakumar, Banadakoppa M. Ramesh

**Affiliations:** 1 HIV Division, Global Health Program, Bill & Melinda Gates Foundation, New Delhi, India; 2 Basic Services, National AIDS Control Organisation, New Delhi, India; 3 India Health Action Trust, Bangalore, India; 4 Independent Consultant, HIV Division, Global Health Program, Bill & Melinda Gates Foundation, New Delhi, India; 5 Karnataka Health Promotion Trust, Bangalore, India; 6 Department of Community Health Sciences, University of Manitoba, Winnipeg, Canada; Baylor College of Medicine, United States of America

## Abstract

**Background:**

Evidence based resource allocation and decentralized planning of an effective HIV/AIDS response requires reliable information on levels and trends of HIV at national and sub-national geographic levels. HIV sentinel surveillance data from antenatal clinics (HSS-ANC) has been an important data source to assess the HIV/AIDS epidemic in India, but has a number of limitations. We assess the value of Prevention of Parent to Child Transmission (PPTCT) programme data to appraise the HIV epidemic in India.

**Methods/Findings:**

HIV data from PPTCT sites were compared to HSS-ANC and general population level surveys at various geographic levels in the states of Karnataka, Maharashtra and Andhra Pradesh. Chi-square tests were used to ascertain statistical significance. PPTCT HIV prevalence was significantly lower than HSS-ANC HIV prevalence (0.92% vs. 1.22% in Andhra Pradesh, 0.65% vs. 0.89% in Karnataka, 0.52% vs. 0.60% in Maharashtra, p<0.001 for all three states). In all three states, HIV prevalence from PPTCT centres that were part of the sentinel surveillance was comparable to HSS-ANC prevalence but significantly higher than PPTCT centres that were not part of the sentinel surveillance. HIV prevalence from PPTCT data was comparable to that from general population surveys. In all three states, significant declines in HIV prevalence between 2007 and 2010 were observed with the PPTCT data set. District level analyses of HIV trends and sub-district level analysis of HIV prevalence were possible using the PPTCT and not the HSS-ANC data sets.

**Conclusion:**

HIV prevalence from PPTCT may be a better proxy for general population prevalence than HSS-ANC. PPTCT data allow for analysis of HIV prevalence and trends at smaller geographic units, which is important for decentralized planning of HIV/AIDS programming. With further improvements to the system, India could replace its HSS-ANC with PPTCT programme data for surveillance.

## Introduction

It is important to base resource allocations for HIV/AIDS programming on sound evidence [1], [2]. Availability of accurate measures of HIV prevalence, incidence and their trends at national, regional and local levels of programming will allow appropriate allocation of resources. Following global standards, India initiated an annual HIV sentinel surveillance (HSS) system in 1992 among antenatal clinic (ANC) attendees to monitor the epidemic throughout the country [3], [4]. The HSS-ANC serves as a warning system for possible epidemic in certain geographic areas [4] and is used to monitor trends in the epidemic [5], [6], and estimate the number of people living with HIV/AIDS (PLHIV) [7]. A subset of ANC clinics is selected to be part of the HSS system, and a sample of women attending these selected sites during a defined time frame are tested for HIV by unlinked anonymous testing (UAT) [8]. Since its inception, the HSS-ANC system has expanded and improved considerably. Compared to 92 HSS-ANC sites in 1998 concentrated mainly in the south, over 676 ANC sites were present in 2010, covering almost all districts of India [9].

There is ample evidence, however, that HSS-ANC has limitations [9]. First, it is not representative of all pregnant women because of the limited sampling frame. Most HSS-ANC sites are located in and around urban district headquarters, and coverage of rural areas, where more than 70% of the population reside [10], is limited. In fact, as in other countries [11], studies in India have shown that HSS-ANC sites overestimate HIV prevalence in the general population [12], [13]. Second, because of the limited number of sites in each district (generally two), analysis of HIV prevalence at the sub-district level is not possible. Third, small sample sizes (only 400–800 women are tested in a given district during a defined sampling period) result in wide confidence intervals and large variations in prevalence between years. As a result, trend analyses at the district level and sub-district level using this data set are not conclusive. Analysis at local levels is critical for HIV programming in a country as large as India. With 27 states and over 600 districts, as well as a heterogeneous HIV epidemic [14], programming must be tailored to fit the local epidemiologic situation [9].

Routinely collected HIV prevalence data from the prevention of mother (or parent) to child transmission programme (PMTCT or PPTCT) provide an additional and expanded source of HIV prevalence data among pregnant women. Advantages of PPTCT data include large sample sizes, lower levels of selection biases at the facility and participant level (if HIV test acceptance levels are high), routine data collection, and low additional cost for data collection. Several countries have explored the utility of PMTCT data for HIV surveillance [15], [16], and some such as Uganda and Thailand have replaced their UAT data with PMTCT data for surveillance due to higher coverage and participation [15].

India launched its PPTCT programme in 2002, and the programme has been considerably expanded since 2007 [17], [18]. In 2010, there were over 8000 PPTCT centres which tested over 6 million women for HIV [19]. Almost all government ANC clinics participate in the programme, and women are routinely tested for HIV during their antenatal visit unless they opt out following pre-test counselling [20]. 96% of the women who were screened at the PPTCT centres accepted to undergo HIV testing. Geographic coverage across the country is also being saturated by making PPTCT services increasingly available at primary health centres, community health centres and in select private facilities through a public-private partnership model.

In the Indian context, Kumar et al. have provided preliminary evidence, through correlations between HIV prevalence from PPTCT and HSS-ANC for the years 2005 to 2007, that supports the use of PPTCT data in place of the annual HIV sentinel surveillance data for determining HIV prevalence and trends [21]. The correlation was high at the state but not district level. While the authors note the small sizes in the HSS-ANC data set as a potential reason for low district level correlation, a more detailed analysis is required to further understand these discrepancies at the district and site levels taking into account the selection bias of HSS-ANC sites as well [22], [23]. Furthermore, the programme has expanded considerably since 2007, and more robust data for the period between 2007 and 2010 are now available for an exhaustive comparative analysis.

Our study addresses three critical questions: (1) Is HIV prevalence data obtained from PPTCT data comparable to HSS-ANC and general populations surveys? (2) If HIV prevalence outcomes are different between data sets, what are plausible reasons, and which data are most reliable? (3) Is there value in using PPTCT data over HSS-ANC data in understanding the progression of the HIV epidemic?

## Methods

### Data Sources, Sample Collection and HIV Testing

#### PPTCT

PPTCT data for period of January 2007 to December 2010 were obtained for r the states of Karnataka, Andhra Pradesh and Maharashtra (three of the four high prevalence states in South India [18]) from the respective State AIDS Control Societies (SACS). The PPTCT data sets include: centre-wise and month-wise data on the number of reporting units, and the ANC attendees counselled, tested and detected HIV positive.

All pregnant women who attend a designated PPTCT ANC clinic are offered counselling and testing to HIV. Unless participants opt out after pre-test counselling, blood samples are tested for HIV using nationally approved diagnostic protocols. Testing and quality control protocols can be found in the national guidelines [20]. Each PPTCT centre reports every month to the SACS, which in turn compile and forward the data to NACO where it is captured in the Central Management and Information System (CMIS).

#### HSS-ANC

The HSS-ANC data set was obtained from the National AIDS Control Organization (NACO) and includes site-wise and year-wise data on the number of sites, number of women tested and number detected HIV positive for all states for the years 2003 to 2008 (the last year for which data were available).

The HSS-ANC data collection is carried out at the designated sentinel sites (subset of PPTCT centres) once a year for 12 weeks. Two sites per district are typically selected: one in the district headquarters and the second in the sub-district headquarters (first referral unit). Consecutive sampling is adopted [8]. A target sample of 400 pregnant women aged 15–49 years attending the antenatal clinic is selected to be part of the HSS during the surveillance period. An eligible pregnant woman can participate in the HSS regardless of the date of her antenatal registration, HIV positivity status (if known), participation in previous rounds of surveillance, or whether she was also tested in a PPTCT centre. Eligible women are tested for HIV using the UAT procedure.

#### General population surveys

The General Population Survey (GPS) is a cross-sectional bio-behavioural survey of the general population conducted in select districts of Karnataka state [9]. The last National Family Health Survey (NFHS-3), a household based survey that captures a wide range of health indicators, was conducted in 2005–06 where a subset of the population in India’s six high prevalence states was also tested for HIV [9].

### Statistical Analysis

To allow for comparison between the PPTCT and HSS-ANC data, the project teams on the ground in the three states determined which centres/sites were designated as PPTCT only, HSS-ANC only, or acted as both (site/centre names in the two data systems were different, which required ground level intelligence to manually sort the data).

The annual, period-specific and geography-specific HIV prevalence rates were computed. PPTCT and HSS-ANC data were compared for the year 2008, as this was the most recent year where data from both HSS-ANC and PPTCT were available. HIV prevalence and 95 percent confidence intervals (CI) were calculated using data from: (1) all HSS-ANC sites; (2) all PPTCT centres; (3) PPTCT centres that were also selected as HSS-ANC sites; and (4) PPTCT centres that were not HSS-ANC sites. The trends in HIV prevalence were computed using only consistent sites/centres across the years.

Also, the HIV prevalence among the women who reported a pregnancy in the two years prior to the survey from the General Population Survey (GPS) in select districts of Karnataka [24]–[26], and the HIV prevalence among the currently pregnant women available from the last National Family Health Survey (NFHS-3) [27]–[29] were compared with the PPTCT data for the corresponding periods and geographies. For comparisons with the NFHS-3 data, PPTCT data from 2005 and 2006 were used for Maharashtra and Andhra Pradesh. For Karnataka state, the 2005 PPTCT data set was not available, so the 2006 PPTCT data set was used. For comparisons with the GPS data, PPTCT data from the two years prior to the GPS were used.

Pearson chi-square tests were used for comparisons across data sources and reference periods, and for trend analysis.

The number of pregnancies in each year was estimated using the projected population for the years 2008, 2009 and 2010 as per the population projections by the Registrar General of India [30] and the crude birth rates (CBR) estimated by the Sample Registration System (SRS) for the years 2008 [31] and 2009 [32]. The 2009 CBR was used to estimate the pregnancies in 2010 since the data for this year were not yet available from the SRS. A pregnancy wastage factor of 15% was considered in the calculation.

### Ethical Approval

This study did not enlist human subjects and did not collect any primary data. All of the secondary data used was originally collected following international ethics norms. Furthermore, the data used have no identifiers associated. Therefore, Institutional Review Ethical Board (IERB) clearances from the institutions involved in the study were not necessary.

Both PPTCT and HSS-ANC data are routinely collected by the Government of India. As per the protocol followed by NACO, a group consent process is followed where the pregnant women have the option of “opting out” from HIV testing [20]. The HSS-ANC sentinel surveillance data are unlinked anonymous and therefore do not require consent to be obtained from the participants [8].

Ethical approval for the GPS in Bagalkot, Belgaum and Mysore districts of Karnataka were granted by the institutional review boards of the Centre Hospitalier Affilié Universitaire de Québec, Québec, Canada; University of Manitoba, Winnipeg, Canada; St John’s Medical College, Bangalore; and the Health Ministry Screening Committee, India [24]–[26].

The NFHS-3 was commissioned by the Ministry of Health and Family Welfare, Government of India. In this survey, all individuals selected in the sample for HIV testing were asked to provide informed voluntary consent to the testing (http://www.nfhsindia.org/nfhs3.html#SURVEY PROCESS).

## Results

### Comparison of the PPTCT and HSS-ANC Data

We compared the data from the two programmes for three southern states – Karnataka, Maharashtra and Andhra Pradesh, since the coverage of pregnant women by PPTCT is much higher in the South, and data could be validated in these three states only (Table 1). Furthermore, the latest HIV sentinel surveillance data available at the time of this analysis were for 2008. Each state had 8–9 times more PPTCT centres, and the number of pregnant women tested was 20–30 times higher compared to the HSS-ANC. In Andhra Pradesh, for example, the PPTCT programme covered a total of 631,926 pregnant women from 612 centres, compared to 20,800 women tested in 69 ANC HIV sentinel sites. On average, each district had 13 PPTCT centres compared to two HSS-ANC sites, and each sub-district had at least one PPTCT centre.

**Table 1:** Comparison of HSS-ANC and PPTCT data sets in three high-prevalence South Indian states, 2008.

	Data set	Data particulars	Andhra Pradesh	Karnataka	Maharashtra
Panel A	HSS-ANC	No. of sites	69	59	82
		No. tested	20800	23192	27940
		No. HIV positive	254	206	167
		% HIV positive	1.22	0.89	0.60
		95% CI	1.07–1.37	0.77–1.01	0.51–0.69
	PPTCT-1: all centres	No. of centres	612	521	640
		No. tested	631926	489614	794392
		No. HIV positive	5833	3167	4107
		% HIV positive	0.92	0.65	0.52
		95% CI	0.90–0.95	0.62–0.67	0.50–0.53
	*p-value (HSS-ANC and PPTCT-1)*		<0.001	<0.001	0.006
Panel B	PPTCT-2: centres whichare also HSS-ANC	No. of centres	70	80	91
		No. tested	161474	141094	186708
		No. HIV positive	1980	1121	1256
		% HIV positive	1.23	0.79	0.67
		95% CI	1.17–1.28	0.75–0.84	0.64–0.71
	*p-value (HSS-ANC and PPTCT-2)*		0.951	0.143	0.487

We also compared HIV prevalence data imputed from the two data sets (Table 1). Due to the large numbers tested, the confidence intervals around the estimates of HIV prevalence were narrower in the PPTCT data compared to the HSS-ANC data. Because all the designated HSS-ANC sites provide PPTCT services, four different comparisons were possible. To determine whether there was a significant difference in HIV prevalence between women tested under the PPTCT and HSS-ANC programmes, we first compared the prevalence of HIV among women tested in all HSS-ANC sites and PPTCT centres (PPTCT-1). In all three states, the prevalence of HIV in the PPTCT data set was significantly lower (p<0.001) compared to HSS-ANC (Table 1 - Panel A). At ANC centres that were selected for sentinel surveillance, we then compared HIV prevalence among women tested under the HSS-ANC with those captured under the PPTCT programme (PPTCT-1). The prevalence of HIV between these two data sets was similar in all states (Table 1 - Panel B). When we compared HIV prevalence from PPTCT centres that were not part of the HSS system (PPTCT-3) with that of HSS-ANC, the prevalence of HIV in all states was significantly lower in the PPTCT-3 data set (p<0.001)). Finally, the prevalence of HIV among pregnant women attending PPTCT centres that were also HSS sites was significantly higher among those attending PPTCT centres that were not HSS-ANC sites in all three states (p<0.001).

### Comparison of HIV Prevalence from PPTCT and General Population Surveys

Table 2 presents district level comparisons between HIV prevalence from the PPTCT data and from the General Population Survey (GPS) conducted over the past few years in three districts in northern Karnataka state (Belgaum, Bellary and Bagalkot). The prevalence of HIV among pregnant women in the GPS was similar to that in the PPTCT data. A similar comparison was conducted using HIV prevalence taken from state level general population surveys (NFHS-3) (Table 3). Again, there was no statistically significant difference in HIV prevalence from the PPTCT data compared to the NFHS-3 data.

**Table 2:** Comparison of PPTCT and general population surveys (GPS) in select districts of Karnataka, 2006–09.

District	GPS		PPTCT		p-value
	Survey year	Percentage HIV positive (N)	Survey year	Percentage HIV positive (N)	
Belgaum	2007	2.92 (306)	2006–07	2.09 (23413)	0.313
Bellary	2007–08	1.74 (335)	2006–07	1.31 (7847)	0.461
Bagalkot*	2009	1.00 (397)	2008–09	2.41 (21728)	0.075

N, total number of individuals tested.

p-value for HIV prevalence comparison.

* Only for Bagalkot, Jamkhandi and Mudhol sub-district areas.

**Table 3:** Comparison of NFHS-3 and PPTCT data in three high-prevalence South Indian states, 2005–06.

State	NFHS-3		PPTCT	p-value
	Total number of women age 15–49 interviewed	Women who were pregnant at time of survey		
		Percentage HIV positive (N)	Percentage HIV positive (N)	
Andhra Pradesh	7128	0.00 (212)	1.50 (647379)	0.074
Karnataka	6008	0.44 (231)	1.98 (110416)	0.096
Maharashtra	9034	0.63 (291)	1.28 (593175)	0.160

N, total number of individuals tested.

p-value for HIV prevalence comparison.

### Geographic Analysis of HIV Prevalence and Trends using PPTCT Data

**Figure 1:**
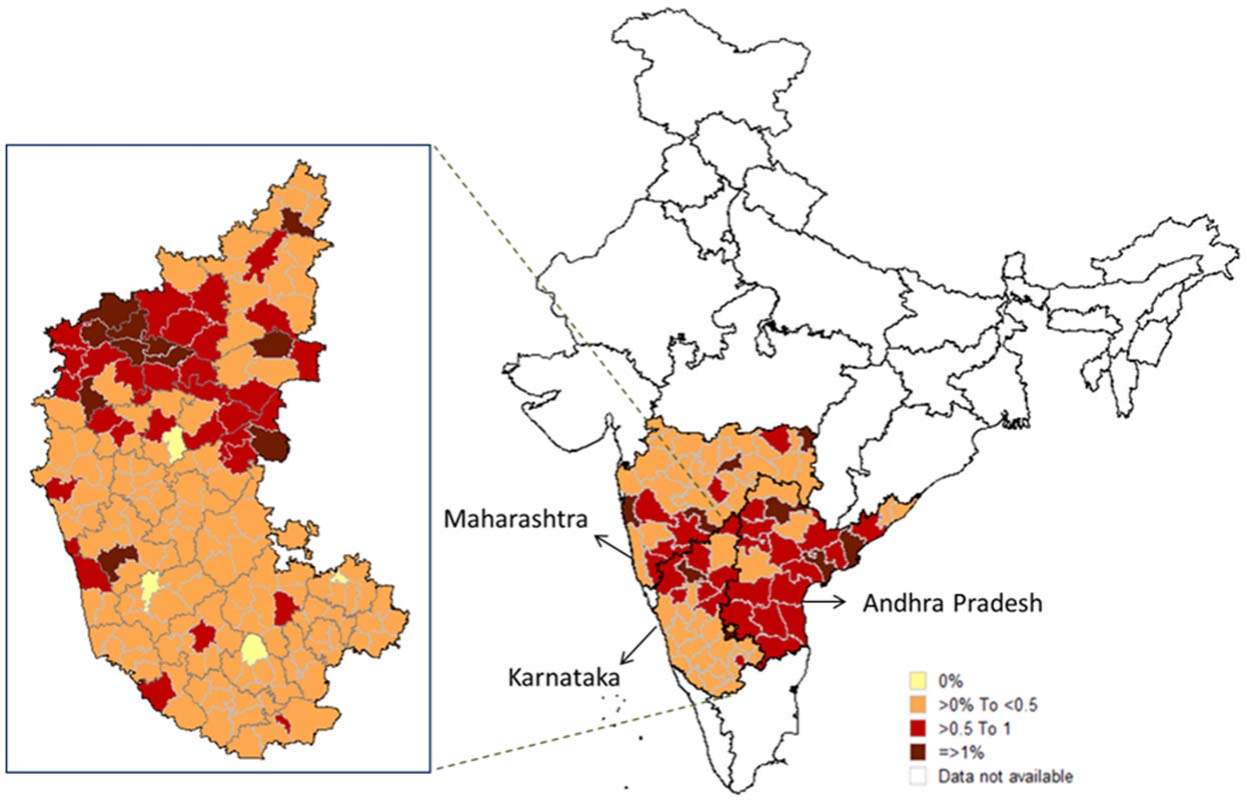
HIV prevalence among ANC women tested in PPTCT centers at district (Andhra Pradesh, Karnataka, Maharashtra, and Tamil Nadu) and sub-district levels (Karnataka), 2009. HIV Prevalence ranges are indicated by color. Lines indicate boundaries at district (graph on right) and sub-district (graph on left) levels.

We explored whether the PPTCT and HSS-ANC data sets could be used for district and sub-district level analyses. On average, each sub-district has one to two PPTCT centres; however, each district has only one or two ANC centres. While district level analysis is possible using both data sets, sub-district level analysis of HIV prevalence was only possible using PPTCT data due to the large numbers of centres and samples. An example for the state of Karnataka in 2009 is shown in Figure 1. Sub-district level analysis revealed further heterogeneity within a district.

**Figure 2:**
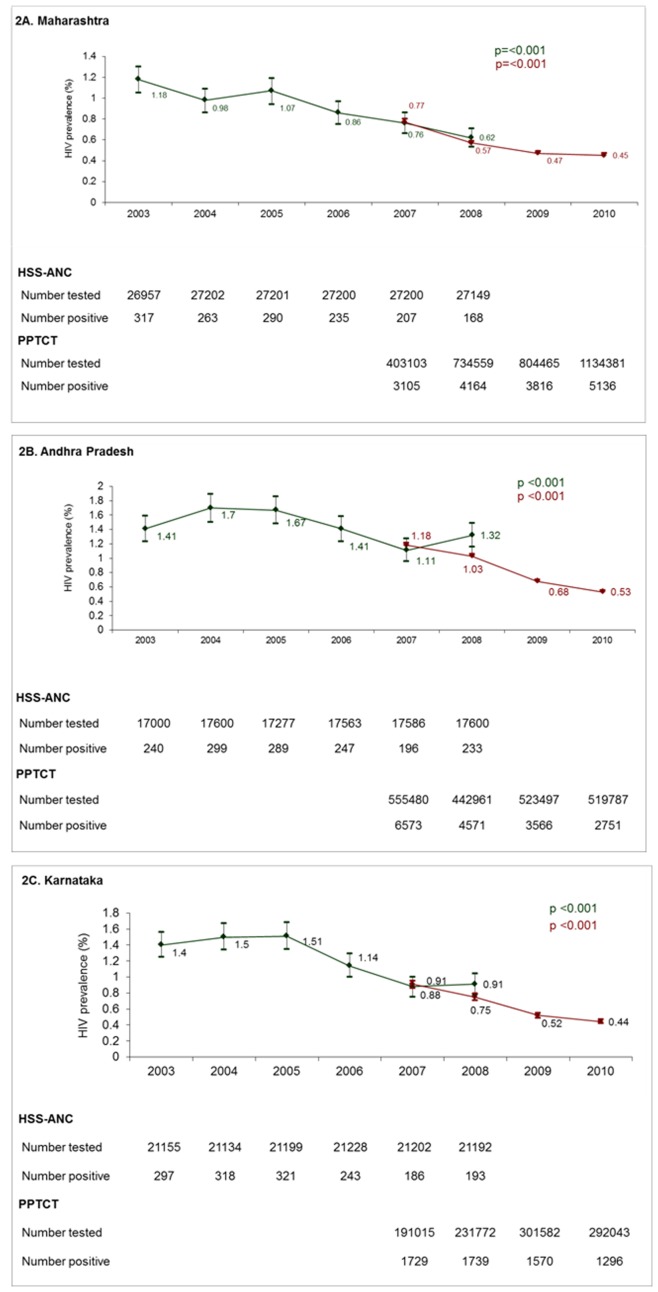
HIV prevalence trends using HSS-ANC and PPTCT data sets. Prevalence of HIV among pregnant women from the HSS-ANC (green lines) and PPTCT data sets (red lines). Diamonds are prevalence (95% CI). P indicates p-value for trend.

The HSS-ANC has been used to examine trends in HIV prevalence at the national, regional and state levels [6]. To determine the feasibility of using the PPTCT data for trend analyses, we conducted trend analyses of HIV prevalence across consistent sites/centres within the HSS-ANC and PPTCT data sets for the years that data were available (Figure 2). In all three states, there were statistically significant declines over time with both data sets. Given that the number of pregnant women tested in the PPTCT programme is comparable between the years, especially from 2008 onwards, these declines likely reflect true declines in prevalence and are not a result of expansion of the PPTCT programme. Unlike the HSS-ANC, district level trends with PPTCT had much narrower confidence intervals, had fewer variations between the years, and did provide conclusive trends over time.

## Discussion

Critical policy level questions are, “What is the utility of the PPTCT data set to appraise the HIV/AIDS epidemic in India?” and, “Should India consider replacing its HIV sentinel surveillance system with the PPTCT programme data to monitor trends and levels of HIV, and if so, when?” We conducted a detailed analysis of the PPTCT data set to compare its utility to HSS-ANC data in appraising the HIV/AIDS epidemic in India. We have built on the preliminary analyses conducted previously by Kumar et al. in three ways: (1) we used data from the time period (2007 to 2010) when the PPTCT programme achieved a high level of scale, including high levels of testing acceptance; (2) we explored the explanations for HIV prevalence outcome differences between PPTCT and HSS-ANC data; and (3) we examined the use of PPTCT data for finer level geographic analysis of HIV prevalence and trends.

We have shown that while HIV prevalence among pregnant women tested at sites common to both the PPTCT and HSS-ANC programmes are comparable, prevalence across all PPTCT centres in a given district or state is significantly lower than prevalence derived from HSS-ANC data. A likely explanation for this is that the PPTCT data set includes centres in peri-urban and rural areas, unlike the HSS-ANC sites, which are largely urban. Prevalence in these HSS-ANC sites is likely to be higher for several reasons. Given that HSS-ANC sites are often found in large district-hospitals, there is a greater chance of getting referral cases of HIV positives in these sites. There is also a bias in the selection of HSS-ANC sites–typically, facilities that do the largest number of deliveries are selected. Furthermore, HIV prevalence in India is likely to be higher in urban areas. Our analysis supports this contention in three ways: first, HIV prevalence in PPTCT centres that are not HSS-ANC sites are lower than in HSS-ANC sites; second, HIV levels between PPTCT centres in non-urban areas are lower than those located in urban areas; and third, HIV prevalence between PPTCT centres and HSS-ANC sites in the same locations are similar.

Our analysis also suggests that HIV prevalence from the PPTCT programme is a better proxy for general population prevalence, as we found the PPTCT prevalence to be statistically comparable to that obtained from several general population surveys. This finding is in line with previous studies, which have suggested that the HSS-ANC overestimates the prevalence of HIV within the general population [12], [13]. Finally, we show that analyses of HIV prevalence at the sub-district level and trends at the district and sub-district level are only possible with PPTCT and not HSS-ANC data.

Our study has several limitations. Individual identifiers, while collected, are not reported in the data sets. Double counting of women in the PPTCT programme is possible, and quality issues with the HSS-ANC data could not be corrected.

In conclusion, we have shown that by using PPTCT data it is possible to estimate HIV prevalence levels and trends over time with extremely tight confidence intervals at the state, district and sub-district levels. Having accurate measures of HIV prevalence at local levels provides HIV/AIDS planners and programmers with data that will allow them to allocate resources more appropriately. However, improvements to the system are needed. In many states, especially in the north, expansion of the HSS-ANC and/or PPTCT programme to better appraise the HIV epidemic would be necessary. Given the large percentage of pregnant women increasingly seeking care in the private sector, providing coverage of PPTCT in these facilities will increase coverage, and make the PPTCT data set more comprehensive. While individual demographic data are collected, they should also be captured in the computerized management information system (CMIS) to allow for stratified analyses, such as age stratification. Finally, more streamlined reporting and better data quality checks to improve the quality of the data set are much needed.

While there are many advantages for using PPTCT data to monitor trends and levels of HIV in India, the full enabling conditions need to be in place. Despite the rapid scale-up of the program since 2007, coverage of pregnant women as per estimated pregnancies remained medium (close to 60% in the high prevalence states) to low (22% in the other states)(see methods) [19]. However, HSS-ANC is even less representative of the pregnant women given that it is a small subset of the PPTCT data set. Low estimated coverage could result from overestimation of pregnancies or from large proportions of unaccounted antenatal care in the private sector or from undocumented home births. According to the District Level Health Survey -III [33], which interviewed 215,000 pregnant women across the country, 75% had received an ANC check-up, of which 55% took place in government facilities, 36% in private facilities, and 10% in the community.

For accurate HIV trend analysis, the PPTCT programme must be scaled up, especially in North India, so that changes in prevalence are not confounded by changes in the profiles of the women being captured. While the PPTCT data set, even in its current state, is a better proxy for population HIV prevalence than the HSS-ANC, increased coverage of pregnant women under the PPTCT programme will strengthen the accuracy of such estimations. Finally, our analysis indicates that the PPTCT data set is most useful from 2007 onwards, therefore limiting the ability to conduct trend analyses for time frames starting before 2007. Given the potential of the PPTCT data set, even with the existing limitations (such as limited expansion of PPTCT services in the North India states), our recommendations are to monitor both the HSS-ANC and the PPTCT data sets for the years to come, use each of their competitive advantages, and conduct a thorough evaluation to assess the full potential of PPTCT for HIV surveillance [34]. The transition from using the HSS-ANC to using the PPTCT data set as a surveillance tool is feasible and advantageous. However, it should be well planned.
